# Changes in Toothbrushing Behaviors Following a Child Dental Care Reform in Israel

**DOI:** 10.3390/children12030289

**Published:** 2025-02-26

**Authors:** Efrat Aflalo, Sharon Barak, Sharon Levi, Lilach Ben Meir, Ariela Giladi, Shlomo Paul Zusman, Yossi Harel Fisch, Miri Shachaf, Moti Zwilling, Riki Tesler

**Affiliations:** 1 Ministry of Aliyah and Integration, Physician Administration & Required Medical Professions, Jerusalem 7178437, Israel; efratbussiba@gmail.com; 2 Department of Nursing, School of Health Sciences, Ariel University, Ariel 40700, Israel; sharoni.baraki@gmail.com; 3 Department of Health Promotion, Ministry of Health, Jerusalem 9101002, Israel; 4 Department of Health Systems Management, School of Health Sciences, Ariel University, Ariel 40700, Israel; 5 Department of Education, Ariel University, Ariel 40700, Israel; 6 Division of Dental Health, Ministry of Health, Jerusalem 9101002, Israel; zusmans@gmail.com; 7 The International Research Program on Adolescent Well-Being and Health, School of Education, Bar-Ilan University, Ramat Gan 52900, Israel; 8 Department of Physical Education, Givat Washington Academic College of Education, Tel Aviv 7923900, Israel; mirile@washington.ac.il; 9 Department of Economics and Business Administration, Ariel University, Ariel 40700, Israel

**Keywords:** oral health, reform, adolescents, toothbrushing behavior

## Abstract

(1) Background: Toothbrushing behavior in children and adolescents is shaped by national dental health policies and sociodemographic and psychological factors. In 2010, child dental care was incorporated into Israel’s National Health Insurance Law (NHIL). This study explored toothbrushing behavior and its predictors before and after this reform. (2) Methods: Data from 36,755 students in grades 6–10 were analyzed from the Health Behaviour in School-Aged Children study conducted pre-reform (1998, 2002, 2006) and post-reform (2010, 2013, 2016). The dependent variable was toothbrushing behavior, while the independent variables included sociodemographic and psychological characteristics. Chi-squared tests compared proportions of compliant toothbrushing behaviors, and logistic regression identified significant predictors. (3) Results: According to recommendations, 59–64% of children brushed their teeth before the reform. This proportion increased significantly post-reform, reaching 73% in 2018. Predictors of compliance included being female, younger, Jewish, and non-observant, having a higher socioeconomic status, and having a better psychological status. These predictors were consistent in pre- and post-reform regression models. (4) Conclusions: The dental care reform positively influenced toothbrushing habits among children and adolescents. However, disparities remain among specific communities. Interventions tailored to address sociodemographic and psychological factors are recommended to enhance regular toothbrushing habits across all populations.

## 1. Introduction

Oral health is part of the general health and well-being of an individual, affecting quality of life [1,2,3]. This understanding gained traction in the early 2000s when oral health was officially recognized as an integral part of health and well-being. Over the years, the term “oral health” has received different interpretations. In 2016, the FDI (World Dental Federation Assembly) proposed a definition that recognizes oral health’s multifaceted nature and characteristics. In fact, the definition states that oral health includes the ability to speak, smile, smell, taste, touch, chew, swallow, and convey emotions through facial expressions confidently and without pain and discomfort [4]. Consequently, the current accepted definition considers oral health as a normal condition of the oral cavity, allowing the individual to eat, smile, and speak without pain or noticeable disease.

There is a high prevalence of oral diseases that cause pain and discomfort and harm the quality of life of many people [3,4,5,6]. One of the most common oral diseases is dental caries, the most common infectious disease in the world. Due to its prevalence, the impact on quality of life, and the resulting economic costs, it is a significant issue for public health that creates a huge burden on individuals, as well as on the entire health system [7,8].

Studies show that the most common oral diseases are preventable by adopting healthy behaviors [3,9,10]. Healthy behavior is defined as the act of promoting, protecting, and maintaining an individual’s health, while health-threatening behavior is associated with a negative impact on health [11]. Correspondingly, there is a relationship between healthy behaviors that maintain oral health and healthy behaviors that maintain general health [11,12]. Research shows that beneficial health behaviors, as well as health-threatening behaviors, exist simultaneously [1,13]. This claim is also true for oral health maintenance behaviors. People who lead an active and healthy lifestyle also pay more attention to oral health [11,12,13]. Brushing teeth at least once a day is considered an important means of maintaining oral health. Toothbrushing is associated with a 2.1-fold lower risk of caries [14,15].

Proper oral hygiene is not the only factor necessary to prevent oral diseases in children [16]. A child’s or adolescent’s biology, lifestyle, and environment are significant factors in maintaining proper oral health [16,17,18]. Oral hygiene was found to be significantly related to common risk behaviors among children and teenagers, such as the consumption of sugary foods and drinks, smoking, and alcohol [19,20,21,22].

In addition, the literature points to various risk factors and differences in behaviors related to maintaining oral health based on sociodemographic variables, such as age, gender, and socioeconomic status [2,8,9,10]. For example, adolescents (defined by the World Health Organization as 10–19) tend to develop their physical self-concept based on physical abilities and appearance [23]. This is of particular importance because maintaining personal hygiene plays a crucial role in self-confidence during this developmental stage. Accordingly, teenagers brush their teeth not for health reasons but to gain self-confidence. In addition, it was observed that 15–16-year-olds have lower plaque levels (which indicates an improvement in oral hygiene) compared to the 11–12-year-old group. Therefore, age may serve as an important sociodemographic variable in maintaining oral health [2,14,17,24].

With respect to gender, girls generally show more interest in oral health and brush their teeth more regularly, but they also consume significantly more sugary foods than boys [2,3,8].

In Israel, the Child Dental Care Reform (CDCR) was implemented in 2010, marking a significant shift in public health policy by integrating pediatric dental care into the national health insurance system [24]. This initiative was aimed not only at improving access to essential dental treatments among children but also at promoting preventive oral health practices [25]. Recognizing that early childhood habits play a crucial role in long-term oral health outcomes, the reform was accompanied by various educational and community-based interventions designed to instill proper brushing techniques, regular dental visits, and awareness of dietary impacts on dental health [26]. These efforts included school-based oral health programs, parental guidance initiatives, nationwide awareness campaigns, and collaborations with dental professionals to ensure the dissemination of best practices [24]. By embedding oral hygiene education within broader health promotion strategies, the CDCR seeks to cultivate lifelong brushing habits among children, reducing the incidence of preventable dental diseases [27]. Specifically, the Health Behaviour in School-aged Children (HBSC) study found that girls aged 11–18 reported brushing their teeth more frequently than boys: about 74% versus about 60%, respectively [28]. Regarding socioeconomic status, it was found that there are significantly more cases of tooth decay among children who grew up in families with a lower socioeconomic background and whose parents have a low income and low level of education [28,29]. It seems that these findings are due to differences in oral hygiene and dietary habits, such as a higher consumption of soft drinks. Correspondingly, brushing teeth twice a day is associated with a higher level of education, especially among mothers, and is also associated with a higher socioeconomic status [8,30].

The adoption of oral health behaviors occurs in childhood and is important for preventing health deterioration at a later age [30,31]. Maintaining oral health from a young age, as one of the behaviors for maintaining body health in general, will prevent the development of oral diseases and affect the course of an individual’s life in various aspects, in the present and in the future [29,30,31]. Moreover, studies have shown that changes in dietary habits and the frequency of toothbrushing are difficult to implement as one grows older if they are not properly assimilated at a younger age [32].

### Dental Health Services in Israel

The National Health Insurance Law) NHIL), which was enacted in Israel in 1994, included a basket of health services. One of the exceptions was dental care, apart from dental services for specific groups, such as oncology patients. Until 2010, almost all dental health services were funded privately, and many individuals had to abstain from oral health treatments due to funding difficulties. The expenses were high compared to OECD countries, and consequently, morbidity was higher [26].

Beginning in July 2010, the CDCR was launched in Israel, with the addition of dental treatments for children and teenagers to the basket of services of NHIL up to the age of 12. These services include preventive and restorative dental treatments [27].

Additionally, school dental services (SDSs) were extended from kindergarten to 9th grade in the whole country. As part of SDSs, health education classes are provided from compulsory kindergarten to the ninth grade, along with a dental examination and the distribution of a toothbrush and toothpaste once a year [33,34]. In fact, with these measures, the Israeli state recognized its responsibility to provide preventive dentistry to the community. In 2013, the reform was extended up to age 14, and in January 2019, the reform was extended up to age 18 [34,35].

The purposes of this study were to (1) explore factors (sociodemographic and psychological characteristics) predicting toothbrushing behavior (brushing teeth more than once/day) among children and adolescents and (2) explore differences in toothbrushing behaviors before and after the dental care reform for children in Israel.

We hypothesized that (1) sociodemographic and psychological characteristics would significantly predict toothbrushing behavior (brushing more than once a day) among children and adolescents, and (2) there would be a significant difference in toothbrushing behavior (brushing more than once a day) before and after the CDCR in Israel.

## 2. Materials and Methods

### 2.1. Study Design

This cross-sectional study investigated changes in toothbrushing behaviors due to the CDCR. The research protocol was approved by the ethics committees of the Israeli Ministry of Education and Bar Ilan University (No. 10203).

### 2.2. Study Procedure

The HBSC study is a cross-national research initiative conducted in collaboration with the World Health Organization (WHO). Established in 1982, the study aims to enhance understanding of adolescents’ health and well-being within their social contexts, including their family, school, and peer environments. The HBSC survey is conducted every four years using a standardized self-report questionnaire administered to students in classrooms. The target age groups are 11-, 13-, and 15-year-olds, representing key developmental stages in adolescence. A clustered sampling design is employed to obtain a representative sample of students within each participating country. Schools are randomly selected, and within each school, classes are chosen to participate in the survey. This approach ensures the diversity and generalizability of the findings. The questionnaire encompasses a wide range of topics related to adolescents’ health behaviors, including oral health, including toothbrushing. It also collects data on demographic variables and social determinants of health.

### 2.3. Study Participants

The study sample consisted of 36,755 children and adolescents in grades 6 through 10 who took part in the Health Behaviour in School-Aged Children survey conducted prior to (1998, 2002, 2006) and following (2010, 2013, 2016) the implementation of the dental care reform.

Students outside the designated study grades (6th, 8th, and 10th) were excluded to ensure consistency in developmental stages. Children and adolescents who had been diagnosed with medical or developmental conditions that significantly affected oral hygiene behaviors (e.g., severe motor impairments, cognitive disabilities) were excluded. Schools that did not fully comply with the Health Behaviour in School-Aged Children (HBSC) survey methodology or had low response rates were excluded from the dataset. Students who did not provide parental consent or chose not to participate in the survey were not included in the study.

### 2.4. Outcome Measures

#### 2.4.1. Dependent Variable—Toothbrushing Frequency

Toothbrushing frequency was assessed with the following question: “How often do you brush your teeth?” Five possible answers were given: (1) “More than once a day”, (2) “Once a day”, (3) “At least once a week but not daily”, (4) “Less than once a week”, and (5) “Never”. This question was dichotomized into “Brushing more than once a day” and “Brushing less frequently”. These two categories were chosen in order to match toothbrushing international recommendations [36].

#### 2.4.2. Independent Variables

*Sociodemographic characteristics*: The study participants reported their self-identified gender (male or female), grade (6th, 8th, or 10th), date of birth, religion (Jewish or non-Jewish), religiousness level (religious, conservative, or non-religious), and socioeconomic status. The last item was evaluated using the first version of the Family Affluence Scale [36,37], which includes the following three items: number of cars, number of vacations, and having own bedroom. Higher scores reflect higher socioeconomic status.

*Psychological characteristics*: Psychological characteristics were assessed using eight questions pertaining to pain (abdominal, headache, back), feelings (anger, bad mood, irritability), sleeping (difficulties falling asleep or sleeping), and dizziness. Each question was scored on a 5-point scale ranging from “almost every day” (1) to “rarely or never” (5). In accordance with the HBSC protocol, two independent scales were created: somatic complaints (headache, abdominal pain, back pain, dizziness) and psychological complaints (anger, bad mood, irritability, difficulty sleeping). For both indexes, higher scores represent better somatic and psychological stats (i.e., fewer symptoms) [36,37,38].

### 2.5. Statistical Analysis

#### 2.5.1. Study Participants’ Sociodemographic and Psychological Characteristics

The descriptive statistics of the study participants’ sociodemographic and psychological characteristics (*n*, percentage, mean, and standard deviation) were calculated for each of the six years separately.

#### 2.5.2. The Proportion of Children Brushing Their Teeth More than Once/Day Before and After the Oral and Dental Health in Children Reform

The percentage of children brushing/not brushing their teeth more than once/day was calculated for each year separately. Next, in each year, differences in prevalence between those brushing/not brushing their teeth at least once/day were examined via chi-square tests. Finally, in order to explore the effects of the reform on toothbrushing behavior, the prevalence of those brushing their teeth more than once/day in each of the post-reform years was compared to those in each of the pre-reform years (i.e., years 2010, 2014, and 2018 vs. years 1998, 2002, and 2006) using chi-squared tests.

#### 2.5.3. Differences in Toothbrushing Frequency According to Sociodemographic and Psychological Characteristics

The prevalence of toothbrushing behaviors was also calculated according to sociodemographic and psychological characteristics. For each characteristic (e.g., males vs. females) in every year separately, differences in the prevalence of those brushing their teeth more than once/day were examined using a chi-squared test (categorical variables) or independent *t*-tests (continuous variables). Differences in prevalence between pre- and post-reform years were also examined with the statistical tests.

The mean changes in the prevalence of those brushing their teeth more than once/day were calculated for the categorical sociodemographic characteristics in the following way: prevalence in years 2010, 2014, or 2018 minus the prevalence in years 1998, 2002, or 2006. A negative or positive score represents a decrease or an increase in the prevalence of those brushing their teeth more than once per day, respectively.

#### 2.5.4. The Prediction of Toothbrushing Habits (More than Once/Day) Before and After the Child Dental Care Reform

Six separate logistic regression analyses were conducted to analyze factors predicting brushing teeth more than once/day. All independent variables were checked for multicollinearity by using the variance of inflation factor (variance of inflation factor >10). As differences in toothbrushing behaviors were observed in all independent variables, they were all included in the regression models.

In all statistical analyses, SPSS Statistics for Windows, version 23 (SPSS Inc., Chicago, IL, USA), was used; the level of significance was set to *p* < 0.05 (2-tailed) [39].

## 3. Results

### 3.1. Population Characteristics

#### Study Participants’ Sociodemographic and Psychological Characteristics

The participants in this study comprised *n* = 36,755 youth who attended the 6th, 8th, and 10th grades during the years 1998 (*n* = 8305), 2002 (*n* = 6143), 2006 (*n* = 6453), 2010 (*n* = 5282), 2014 (*n* = 7195), and 2018 (*n* = 3377). The first and last three years are before and after the oral and dental health in children reform, respectively. For an additional description of the study participants, refer to Table 1.

### 3.2. The Proportion of Children Brushing Their Teeth More than Once/Day Before and After the Child Dental Care Reform

**Figure 1:**
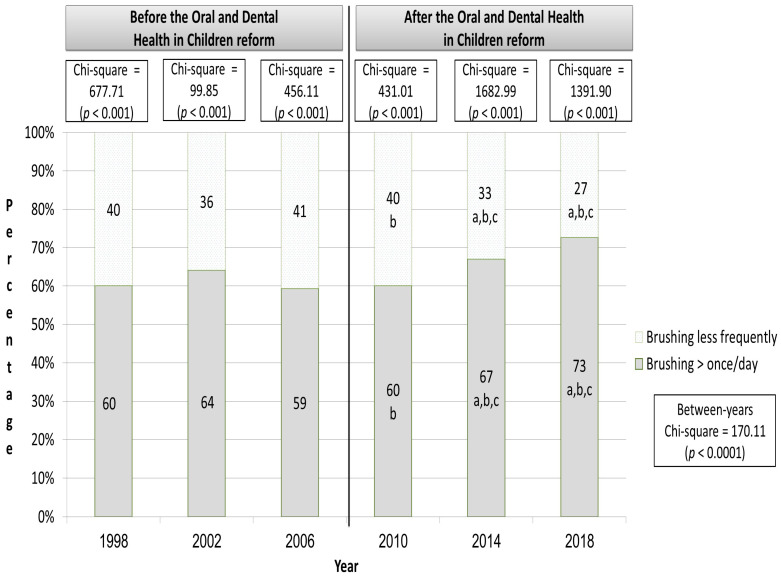
The proportion of children brushing their teeth more than once a day according to years before and after the Child Dental Care Reform. Notes: ^a^, the proportion is statistically significantly different from the year 1998 (*p* < 0.005, 2-tailed); ^b^, the proportion is statistically significantly different from that in the year 2002 (*p* < 0.005, 2-tailed); ^c^, the proportion is statistically significantly different from that in the year 2006 (*p* < 0.005, 2-tailed).

In all study years, the proportion of children brushing their teeth more than once/day was statistically significantly greater than that of children brushing their teeth less frequently (chi-square range: 99.85 to 1682; *p* < 0.001). In the first year of the reform (2010), the proportion of children brushing their teeth more than once/day was statistically significantly smaller than that in 2002 (60 vs. 64%, respectively). However, in post-reform years 2014 and 2018, the proportion of children brushing their teeth more than once/day was statistically significantly greater than that observed in the three pre-reform years. More specifically, in 2014, the prevalence gap ranged from three to eight percent, while in 2018, the gap increased and ranged from nine to fourteen percent (Figure 1).

### 3.3. Differences in Toothbrushing Frequency According to Sociodemographic and Psychological Characteristics

In all study years (both before and after the reform), the prevalence of female, Jewish, and non-religious children brushing their teeth more than once/day was statistically significantly greater than that of male, non-Jewish, religious, and conservative children. Moreover, in comparison to children not brushing their teeth frequently enough, children brushing their teeth more than once/day were statistically significantly younger, were from a higher socioeconomic status, and presented fewer somatic (except for the year 2002) and psychological complaints (Table 2).

### 3.4. Differences in Toothbrushing Frequency According to Years Before and After the Child Dental Care Reform

In comparison to the three pre-reform years evaluated, in post-reform years 2014 and 2018, the proportion of female, male, Jewish, non-Jewish, conservative, and non-religious children brushing their teeth more than once/day was statistically significantly greater. In addition, in the post-reform years, children brushing their teeth more than once/day were statistically significantly younger than the observed ages in the three pre-reform years. No differences between the pre- and post-reform years were observed in socioeconomic status and somatic and psychological complaints (*p* > 0.05). For additional information, refer to Table 2.

Overall, in comparison to pre-reform years, in 2010, no constant improvement was observed in toothbrushing habits. More specifically, a decrease in toothbrushing was observed in both males and females and Jewish and non-Jewish children, as well as in children with various religiousness levels. In 2014, a decrease in toothbrushing habits was observed only in one instance (religious children—a decrease of 0.11% in comparison to 2002). In this year, toothbrushing habits increased by approximately 2% (in non-religious in comparison to 2002) to 10% (in non-religious in comparison to 1998 and non-Jewish in comparison to 2006). Finally, in 2018, in all subgroups analyzed, except for non-Jewish (in comparison to years 1998 and 2002), males (in comparison to 2002), and religious and conservative children (in comparison to 2002), there was an increase of >10% in toothbrushing habits (Table 3).

### 3.5. The Prediction of Toothbrushing Habits (More than Once/Day) Before and After the Child Dental Care Reform

In all pre-reform years, sex (being female), religion (being Jewish), socioeconomic status (higher status), and psychological complaints (fewer psychological complaints) statistically significantly predicted brushing teeth more than once/day. Age (younger age) and being non-religious in comparison to religious also predicted brushing teeth more than once/day in at least one of the three pre-reform years. Overall, the models explained five (year 2002) to seven (year 2006) percent of the variability in toothbrushing behavior (chi-square range: 254.41 to 361.08; *p* < 0.0001). For additional information, refer to Table 4.

Overall, similar results were observed in the post-reform regression models, with models explaining five (year 2010) to seven (years 2014 and 2018) percent of the variability in toothbrushing behavior (chi-square range: 166.43 to 328.73; *p* < 0.0001). For additional information, refer to Table 5.

## 4. Discussion

This study aimed to explore the predictors of toothbrushing behavior among children and adolescents and examine changes in toothbrushing frequency before and after the implementation of the Child Dental Care Reform in Israel. Regarding our first hypothesis, our findings support the rejection of the null, as sociodemographic and psychological characteristics were found to significantly predict toothbrushing behavior. Specifically, being female, younger, Jewish, and non-observant, having a higher socioeconomic status, and reporting better psychological well-being were positively associated with brushing teeth more than once a day. Similarly, we were able to reject the null for the second hypothesis, as significant differences were observed in toothbrushing behavior before and after the reform. The proportion of children brushing their teeth more than once a day increased substantially post-reform, suggesting that the Child Dental Care Reform had a positive impact on oral hygiene practices among school-aged children. These results highlight the role of public health policy in shaping preventive behaviors and emphasize the need for targeted interventions to address disparities observed among specific demographic groups.

This study’s purpose was to investigate the sociodemographic and psychological factors associated with toothbrushing habits among adolescents while also examining variations in toothbrushing frequencies before and after the implementation of the Child Dental Care Reform in Israel. The study results indicate a positive impact of the policy change on toothbrushing behaviors, with a significant increase of over 10% in the proportion of adolescents brushing their teeth more than once a day in 2018 as compared to pre-reform years. Moreover, this increase in toothbrushing frequency was observed across all studied sociodemographic groups following the implementation of the reform.

Across all examined years, a larger proportion of children adhered to the recommended toothbrushing frequency in comparison to those who did not conform. In both the years preceding the reform and following it, adhering to toothbrushing recommendations was correlated with sociodemographic factors such as gender (female), religious identity (Jewish), non-religious affiliation, younger age, and higher socioeconomic status. Additionally, a higher level of psychological well-being was linked to an increased frequency of toothbrushing.

These results corroborate earlier research that identified socioeconomic status and lower levels of maternal education as contributing factors to an elevated caries risk and poor dental health [2,9,10]. Similarly, research has shown that girls exhibit improved oral health outcomes [9,12,27] and are inclined to maintain more consistent toothbrushing practices as compared to boys [28,29]. Moreover, the outcomes of the 2017/18 HBSC study also indicate that the frequency of adhering to the recommended toothbrushing routine is higher among girls and adolescents from more affluent households [29].

An interesting finding of the present study pertains to age. Our findings consistently indicated that younger adolescents demonstrated a higher likelihood of brushing their teeth more than once a day, in contrast to previous research suggesting an improvement in oral hygiene among 15–16-year-olds as compared to 11–12-year-olds [3,40]. Findings extracted from the 2017/2018 HBSC study reveal a divergent pattern across certain countries, where a mixed tendency was observed [2,29]. While older boys demonstrated a decreased propensity to brush their teeth twice daily in comparison to their younger peers, a contradictory trend emerged among girls. Moreover, as adolescents mature, they may progressively engage in behaviors that compromise their health, such as skipping breakfast [41] and experiencing a decrease in high levels of physical activity [17,28]. This suggests that as age may serve as a significant sociodemographic factor in maintaining oral health, other variables linked to adolescents’ cultural environment and lifestyle must be included in order to gain a comprehensive understanding [2,20,21].

Religion and religiosity also emerge as noteworthy factors influencing toothbrushing behavior in this study. Former research also found that the improvement in oral health outcomes within the Jewish sector following the reform was greater than that in the Arab sector [1,10,27,34]. The literature presents a mixed array of findings concerning the interplay between religion, religiosity, and health outcomes. A recent multi-national comparative study suggests that the relationship between religiosity and health is complicated, influenced in part by geopolitical and broader macropsychosocial factors [1,10,34]. Notably, in Israel, religion and the level of religiosity have been associated with patterns in preventive medical procedures. For instance, Pinchas-Mizrachi et al. (2021) observed a lower tendency toward mammography screening among Israeli Arab women and among religious as well as conservative Jewish women as compared to secular Jewish women [42]. Moreover, religion and religiosity may also bear connections to socioeconomic status, as both Israeli Arabs and religious Jews have more children and lower income as compared to non-religious Jews [42]. Jews and Arabs, as well as religious and non-religious Jews, often inhabit distinct communities, study in different state schools, and may share comparable cultural and lifestyle attributes [24,42,43], potentially influencing health behaviors.

Another finding pertains to the effect of psychological well-being on toothbrushing frequency. A wealth of evidence underscores the connection between psychological well-being and reduced risks of disease and mortality [2,8,35]. A positive psychological state exhibits an inverse correlation with risky behaviors, such as smoking, alcohol consumption, and substance abuse [23,28,44]. These risky behaviors are associated with irregular toothbrushing habits, whereas consistent brushers often embrace health-promoting activities, such as sports participation [18,20,21]. Furthermore, dental problems are linked to poor emotional well-being [1,2,3]. Together, these observations imply that, similarly to other health outcomes, dental health may hold a bidirectional relationship with emotional well-being.

The strengths of this study lie in its substantial sample size and the span of years under examination. Furthermore, its significant findings have the potential to provide insights and recommendations relevant to the domains of oral health policy, health education, and well-being among adolescents in Israel.

Nevertheless, certain limitations should be acknowledged. Firstly, all study variables were based on self-report measures, which could potentially introduce common method biases. Secondly, the adoption of a cross-sectional design prevents the establishment of causal relationships, especially in relation to the association between toothbrushing and psychological well-being. Additionally, there is a possibility that factors other than the 2010 reform might have influenced the observed positive increase in toothbrushing frequency. Future studies could blend self-report measures with existing medical data concerning adolescent oral health and employ a mixed-method design to achieve a deeper comprehension of the factors influencing oral hygiene among adolescents. Employing a longitudinal design in the future could offer a clearer portrayal of the directions of these relationships. With regard to the CDCR in Israel, this study shows the importance of promoting healthy toothbrushing habits among children through the implementation of supervised toothbrushing programs in educational settings. These programs, initiated in kindergarten, involve daily supervised brushing sessions using fluoride toothpaste while effectively integrating oral hygiene practices into the children’s daily routines. This study’s findings indicate that such supervised programs not only improve children’s brushing habits but also enhance their overall dental health. For instance, a previous study evaluating a two-year supervised toothbrushing program in Southern Israel found that, among participating children, there were better dental health outcomes compared to non-participants, with fewer carious teeth and a higher number of treated carious lesions [24]. By embedding these practices within the educational framework, the CDCR ensures that children develop consistent brushing habits from an early age, leading to improved oral health outcomes in young children.

Moreover, the longitudinal analysis of toothbrushing habits among children and adolescents from 1998 to 2018 encompasses a substantial timeframe, during which various socioeconomic factors may have significantly influenced oral hygiene behaviors. The academic literature indicates that higher socioeconomic status is associated with an increased prevalence of regular toothbrushing among adolescents; a previous study analyzing data from 20 countries between 1994 and 2018 found that adolescents from more affluent families were more likely to brush their teeth regularly [2].

Additionally, disparities in oral hygiene practices have been observed among different socioeconomic groups within the same country. A study comparing oral hygiene habits among preschool children from low socioeconomic neighborhoods in Israel found that children of native Israeli parents were more likely to brush their teeth at least once daily compared to children of Ethiopian immigrant parents [25]. These findings suggest that factors such as family income, parental education, and cultural background can significantly impact children’s toothbrushing habits. Therefore, when evaluating changes in oral hygiene behaviors over extended periods, it is crucial to consider the influence of socioeconomic determinants to accurately assess the effectiveness of public health interventions and to identify areas requiring targeted efforts to reduce health disparities [9,25,28]. The findings indicate that the Child Dental Care Reform improved dental hygiene habits among children and adolescents; however, significant disparities persist among the different communities in Israel. These differences highlight the need for targeted public health interventions that address the unique barriers faced by specific demographic groups, ensuring equitable access to preventive dental care and oral health education.

## 5. Conclusions

The outcomes of the current study emphasize the effectiveness of the Child Dental Care Reform in improving students’ oral hygiene practices. Hence, we advocate for the sustained implementation of policy measures at both public and educational levels to enhance dental care accessibility and literacy, particularly among non-Jewish, religious, and economically disadvantaged subgroups. Moreover, policy interventions should accentuate educational initiatives targeted at at-risk adolescents, given their association with poor oral health and less consistent toothbrushing habits. Similarly, heightened consideration should be directed toward adolescent boys throughout their maturation, as the findings highlight a propensity to compromise oral health behaviors during the later stages of adolescence.

To reduce disparities in oral hygiene habits, targeted interventions should focus on culturally adapted oral health education programs, increased accessibility to preventive dental services in underserved communities, and school-based initiatives promoting daily toothbrushing habits. Future research should explore integrating psychological support strategies and community engagement efforts to address behavioral barriers and enhance long-term adherence to proper oral hygiene practices across diverse sociodemographic groups.

## Figures and Tables

**Table 1:** Study participants’ sociodemographic and psychological characteristics (*n* = 36,755).

Characteristics		Before Child Dental Care Reform			After Child Dental Care Reform		
		Year 1998 (*n* = 8305)	Year 2002 (*n* = 6143)	Year 2006 (*n* = 6453)	Year 2010 (*n* = 5282)	Year 2014 (*n* = 7195)	Year 2018 (*n* = 3377)
**Sex, *n* (%)**	Females	4103 (49.40)	3277 (53.34)	3665 (56.79)	2780 (52.63)	3659 (50.85)	1850 (54.78)
	Males	4202 (50.59)	2866 (46.65)	2788 (43.20)	2502 (47.36)	3536 (49.14)	1527 (45.21)
**Grade, *n* (%)**	6th	3798 (45.73)	2007 (32.67)	2014 (31.21)	1881 (35.61)	3130 (43.50)	1177 (34.85)
	8th	2245 (27.03)	2418 (39.36)	2078 (32.20)	1722 (32.60)	2324 (32.30)	1056 (31.27)
	10th	2262 (27.23)	2418 (39.36)	2361 (36.58)	1679 (31.78)	1741 (24.19)	1144 (33.87)
**Age, mean (SD)**		13.80 (1.55)	14.05 (1.64)	14.28 (1.63)	13.11 (1.73)	13.87 (1.66)	13.31 (1.65)
**Religion, *n* (%)**	Jewish	5216 (62.80)	4608 (75.01)	3963 (61.41)	3698 (70.01)	5159 (71.70)	2199 (65.11)
	Non-Jewish	3089 (37.79)	1535 (24.98)	2490 (38.58)	1584 (29.98)	2036 (28.29)	1178 (34.88)
**Religiousness level, *n* (%)**	Religious	1827 (21.99)	1228 (19.99)	1639 (25.39)	1431 (27.09)	2352 (32.68)	634 (18.77)
	Conservative	3728 (44.88)	2770 (45.09)	2903 (44.98)	2181 (41.29)	2654 (36.88)	1502 (44.47)
	Non-religious	2750 (33.11)	2145 (34.91)	1911 (29.61)	1670 (31.61)	2189 (30.42)	1241 (36.74)
**Socioeconomic status, mean (SD)**		6.32 (1.43)	6.34 (1.44)	6.67 (1.63)	6.89 (1.61)	5.75 (1.50)	6.34 (1.54)
**Somatic complaints, mean (SD)**		14.97 (4.05)	15.59 (3.78)	14.76 (4.48)	15.35 (4.38)	15.24 (4.13)	15.21 (4.37)
**Psychological complaints, mean (SD)**		13.63 (4.32)	13.64 (4.20)	13.05 (4.76)	14.03 (4.75)	13.53 (4.61)	13.36 (4.73)

Notes: SD, standard deviation.

**Table 2:** Differences in sociodemographic and psychological characteristics according to toothbrushing status before and after the Child Dental Care Reform.

Characteristics		Year 1998		Year 2002		Year 2006		Year 2010		Year 2014		Year 2018	
		More 1/Day	Less Frequently	More 1/Day	Less Frequently	More 1/Day	Less Frequently	More 1/Day	Less Frequently	More 1/Day	Less Frequently	More 1/Day	Less Frequently
**Sex, %**	Females	68.13	31.87	71.23	28.77	64.89	35.11	65.73 ^a,b^	34.27	73.81 ^a,c^	26.19	78.84 ^a–c^	34.79
	Males	51.92	48.08	56.24	43.76	52.30	47.70	53.75 ^b^	46.25	60.36 ^a–c^	39.64	65.21 ^a–c^	21.16
	χ^2^ *(p*-value)	248.74 (<0.0001)		149.28 (<0.0001)		94.11 (<0.0001)		78.56 (<0.0001)		136.62 (<0.0001)		68.47 (<0.0001)	
**Age, years: mean (SD)**		13.77 (1.55)	13.85 (1.56)	13.99 (1.62)	14.17 (1.66)	14.20 (1.68)	14.40 (1.67)	13.02 (1.71) ^a–c^	13.24 (1.75)	13.72 (1.63) ^b,c^	13.85 (1.67)	13.29 (1.69) ^a–c^	13.49 (1.79)
Statistic *t* (*p*-value)		−2.29 (0.02)		−3.88 (0.0001)		−4.65 (<0.0001)		−4.53 (<0.0001)		−3.17 (0.001)		−3.03 (0.002)	
**Religion, %**	Jewish	63.31	36.69	66.54	33.46	66.32	33.68	61.48 ^b,c^	38.52	70.73 ^a–c^	29.27	77.22 ^a–c^	22.78
	Non-Jewish	54.96	45.05	56.33	43.67	48.41	51.59	57.15 ^c^	42.85	58.08 ^a,c^	41.92	64.39 ^a–c^	35.61
	χ^2^ *(p*-value)	65.28 (<0.0001)		49.67 (<0.0001)		204.87 (<0.0001)		7.38 (0.006)		94.49 (<0.0001)		64.95 (<0.0001)	
**Religiousness level, %**	Religious	56.91 ^e,f^	43.09	62.32 ^f^	37.68	53.37 ^e,f^	46.63	49.68 ^a–c,e,f^	50.32	62.21 ^a,c,e,f^	37.79	68.44 ^a–c,f^	31.56
	Conservative	59.54 ^d,f^	40.46	63.65 ^f^	36.35	58.98 ^d,f^	41.02	62.63 ^a,c,d,f^	37.37	66.97 ^a–c,d,f^	33.03	70.81 ^a–c,f^	29.19
	Non-religious	62.84 ^d,e^	37.16	66.13 ^d,e^	33.87	65.91 ^d,e^	34.09	65.76 ^a,d,e^	34.24	72.78 ^a–c,d,e^	27.22	77.23 ^a–c,d,e^	22.77
	χ^2^ *(p*-value)	16.17 (0.0003)		5.63 (0.05)		57.44 (<0.0001)		95.43 (<0.0001)		29.76 (<0.0001)		21.23 (<0.0001)	
**Socioeconomic status, mean (SD)**		6.42 (1.43)	6.16 (1.42)	6.44 (1.41)	6.14 (1.48)	6.88 (1.58)	6.37 (1.64)	7.01 (1.58)	6.70 (1.63)	5.85 (1.49)	5.50 (1.46)	6.56 (1.53)	6.20 (1.53)
Statistic *t* (*p*-value)		−8.04 (<0.0001)		−7.91 (<0.0001)		−12.27 (<0.0001)		−6.87 (<0.0001)		−9.11 (<0.0001)		−6.16 (<0.0001)	
**Somatic complaints, mean (SD)**		15.11 (3.95)	14.87 (4.11)	15.65 (3.71)	15.48 (3.88)	14.92 (4.43)	14.55 (4.51)	15.48 (4.33)	15.14 (4.46)	15.37 (4.10)	15.04 (4.20)	15.55 (4.30)	14.67 (4.57)
Statistic *t* (*p*-value)		−2.51 (0.01)		−1.59 (0.11)		−3.02 (0.002)		−2.64 (0.008)		−2.80 (0.005)		−5.21 (<0.0001)	
**Psychological complaints, mean (SD)**		13.87 (4.30)	13.32 (4.30)	13.84 (4.17)	13.28 (4.22)	13.83 (4.16)	13.18 (4.12)	14.37 (4.73)	14.26 (4.73)	13.73 (4.61)	13.17 (4.56)	13.91 (4.70)	13.29 (4.62)
Statistic *t* (*p*-value)		−5.45 (<0.0001)		−4.86 (<0.0001)		−4.85 (<0.0001)		−5.87 (<0.0001)		−4.27 (<0.0001)		−3.43 (0.0006)	

Notes: ^a^, significantly different from 1998 (*p* < 0.005); ^b^, significantly different from 2002 (*p* < 0.005); ^c^, significantly different from 2006 (*p* < 0.005); ^d^, significantly different from religious (*p* < 0.005); ^e^, significantly different from conservative (*p* < 0.005); ^f^, significantly different from non-religious (*p* < 0.005); SD, standard deviation.

**Table 3:** Mean changes in the percentage of participants brushing their teeth more once/day (*n* = 36,755) during the Child Dental Care Reform.

Characteristics		Year 2010			Year 2014			Year 2018		
		2010 Minus 1998: %	2010 Minus 2002: %	2010 Minus 2006: %	2014 Minus 1998: %	2014 Minus 2002: %	2014 Minus 2006: %	2018 Minus 1998: %	2018 Minus 2002: %	2018 Minus 2006: %
**Sex**	Females	−2.4	−5.50	0.84	5.68	2.58	8.92	10.71	7.61	13.95
	Males	1.83	−2.49	1.45	8.44	4.12	8.06	13.29	8.97	12.91
**Religion**	Jewish	−1.83	−5.06	−4.84	7.42	4.19	4.41	13.91	10.68	10.9
	Non-Jewish	2.19	0.82	8.74	3.12	1.75	9.67	9.43	8.06	15.98
**Religiousness level**	Religious	−7.23	−12.64	−3.69	5.3	−0.11	8.84	11.53	6.12	15.07
	Conservative	3.09	−1.02	3.65	7.43	3.32	7.99	11.27	7.16	11.83
	Non-religious	2.92	−0.37	−0.15	9.94	6.65	6.87	14.39	11.11	11.32

**Table 4:** The prediction of toothbrushing habits (brushing more than once/day) before the Child Dental Care Reform.

	Variables	Coefficient	Standard Err	Wald	Odds Ratio	95% CI	*p*
**1998**	Constant	−1.32	0.29	20.93	-	-	<0.001
	Sex, in comparison to males	0.75	0.05	218.95	2.11	1.91–2.33	<0.001
	Age, years	−0.009	0.01	0.31	0.99	0.95–1.02	0.57
	Religion, in comparison to non-Jewish	0.34	0.05	39.70	1.41	1.27–1.57	<0.001
	Religiousness level—conservative, in comparison to religious	0.06	0.06	1.12	1.07	0.94–1.21	0.28
	Religiousness level—non-religious, in comparison to religious	0.15	0.07	4.69	1.16	1.01–1.34	0.03
	Socioeconomic status, score	0.14	0.01	62.40	1.15	1.11–1.19	<0.001
	Somatic complaints, score	−0.002	0.007	0.14	0.99	0.98–1.01	0.70
	Psychological complaints, score	0.02	0.007	15.19	1.02	1.01–1.04	0.0001
	Model summary	Nagelkerke R^2^ = 0.06; χ^2^ = 361.08; *p* = <0.0001					
**2002**	Constant	−0.32	0.37	0.76	-	-	0.38
	Sex, in comparison to males	0.72	0.06	143.36	2.06	1.83–2.33	<0.001
	Age, years	−0.05	0.01	8.00	0.94	0.91–0.98	0.04
	Religion, in comparison to non-Jewish	0.40	0.07	30.41	1.49	1.29–1.72	<0.001
	Religiousness level—conservative, in comparison to religious	0.03	0.08	0.14	1.03	0.88–1.20	0.70
	Religiousness level—non-religious, in comparison to religious	0.20	0.08	5.98	1.23	1.04–1.45	0.01
	Socioeconomic status, score	0.14	0.02	45.93	1.15	1.10–1.20	<0.001
	Somatic complaints, score	0.01	0.008	2.30	1.01	0.99–1.02	0.12
	Psychological complaints, score	−0.03	0.008	13.93	1.03	1.01–1.04	0.0002
	Model summary	Nagelkerke R^2^ = 0.05; χ^2^ = 254.41; *p* = <0.0001					
**2006**	Constant	−1.03	0.31	10.65	-	-	0.001
	Sex, in comparison to males	0.54	0.06	77.96	1.71	1.52–1.93	<0.001
	Age, years	−0.04	0.01	7.23	0.95	0.91–0.98	0.007
	Religion, in comparison to non-Jewish	0.60	0.06	81.10	1.83	1.60–2.09	<0.001
	Religiousness level—conservative, in comparison to religious	0.13	0.07	3.16	1.14	0.98–1.32	0.07
	Religiousness level—non-religious, in comparison to religious	0.14	0.08	2.91	1.15	0.97–1.36	0.08
	Socioeconomic status, score	0.17	0.01	87.21	1.19	1.15–1.24	<0.001
	Somatic complaints, score	−0.005	0.008	0.33	0.99	0.97–1.01	0.56
	Psychological complaints, score	0.01	0.008	4.64	1.01	1.00–1.03	0.03
	Model summary	Nagelkerke R^2^ = 0.07; χ^2^ = 328.73; *p* = <0.0001					

**Table 5:** The prediction of toothbrushing habits (brushing more than once/day) after the Child Dental Care Reform.

	Variables	Coefficient	Standard Err	Wald	Odds Ratio	95% CI	*p*
**2010**	Constant	−1.57	0.35	19.42	-	-	<0.001
	Sex, in comparison to males	0.51	0.06	57.49	1.67	1.46–1.91	<0.001
	Age, years	0.01	0.02	0.55	1.01	0.97–1.05	0.45
	Religion, in comparison to non-Jewish	0.002	0.07	0.001	1.00	0.86–1.16	0.97
	Religiousness level—conservative, in comparison to religious	0.42	0.08	25.80	1.52	1.29–1.79	<0.001
	Religiousness level—non-religious, in comparison to religious	0.48	0.09	28.53	1.62	1.36–1.94	<0.001
	Socioeconomic status, score	0.12	0.02	32.71	1.13	1.08–1.18	<0.001
	Somatic complaints, score	−0.01	0.01	1.67	0.98	0.96–1.00	0.19
	Psychological complaints, score	0.04	0.009	25.20	1.04	1.03–1.06	<0.001
	Model summary	Nagelkerke R^2^ = 0.05; χ^2^ = 166.43; *p* = <0.0001					
**2014**	Constant	−1.08	0.32	10.89	-	-	0.001
	Sex, in comparison to males	0.76	0.06	149.88	2.15	1.90–2.43	<0.001
	Age, years	−0.03	0.01	3.92	0.96	0.92–0.99	0.04
	Religion, in comparison to non-Jewish	0.64	0.06	88.19	1.90	1.66–2.18	<0.001
	Religiousness level—conservative, in comparison to religious	0.04	0.08	0.24	1.04	0.88–1.21	0.61
	Religiousness level—non-religious, in comparison to religious	0.11	0.09	1.58	1.12	0.93–1.34	0.20
	Socioeconomic status, score	0.18	0.02	73.13	1.20	1.15–1.25	<0.001
	Somatic complaints, score	−0.004	0.009	0.19	0.99	0.97–1.01	0.66
	Psychological complaints, score	0.03	0.008	17.39	1.03	1.01–1.05	<0.001
	Model summary	Nagelkerke R^2^ = 0.07; χ^2^ = 328.73; *p* = <0.0001					
**2018**	Constant	−0.17	0.38	0.21	-	-	0.64
	Sex, in comparison to males	0.78	0.08	94.13	2.19	1.87–2.56	<0.001
	Age, years	−0.09	0.02	15.54	0.91	0.87–0.95	0.0001
	Religion, in comparison to non-Jewish	0.64	0.09	46.98	1.89	1.58–2.28	<0.001
	Religiousness level—conservative, in comparison to religious	0.15	0.10	2.06	1.17	0.94–1.42	0.15
	Religiousness level—non-religious, in comparison to religious	0.18	0.11	2.49	1.20	0.95–1.51	0.11
	Socioeconomic status, score	0.13	0.02	26.60	1.14	1.08–1.20	<0.001
	Somatic complaints, score	0.02	0.01	4.69	1.02	1.00–1.05	0.03
	Psychological complaints, score	0.01	0.01	1.34	1.01	0.99–1.03	0.24
	Model summary	Nagelkerke R^2^ = 0.07; χ^2^ = 220.50; *p* = <0.0001					

## Data Availability

The raw data supporting the conclusions of this article will be made available by the authors upon request due to ethical reasons.
